# *L. plantarum *prevents *Enteroinvasive Escherichia coli*-induced tight junction proteins changes in intestinal epithelial cells

**DOI:** 10.1186/1471-2180-9-63

**Published:** 2009-03-31

**Authors:** Huanlong Qin, Zhongwei Zhang, Xiaomin Hang, Yanqun Jiang

**Affiliations:** 1 Department of Surgery, Affiliated Sixth People's Hospital, Shanghai Jiao Tong University, Shanghai 200233, PR China

## Abstract

**Background:**

It is increasingly recognized that *Lactobacillus plantarum (L. plantarum) *has the ability to protect against Enteropathogenic *Escherichia coli *(EPEC)-induced damage of the epithelial monolayer barrier function by preventing changes in host cell morphology, attaching/effacing (A/E) lesion formation, monolayer resistance, and macromolecular permeability. However, the cellular mechanism involved in this protective effect still remained to be clarified.

**Methods:**

This study was to investigate the effect of *L. plantarum *on the changes of Caco-2 cells responding to Enteroinvasive Escherichia coli (EIEC), the permeability of cell monolayer and the transmissivity of dextran, and the distribution and expression of the tight junction (TJ) proteins, such as Claudin-1, Occludin, JAM-1 and ZO-1 were examined when infected with EIEC or adhesived of *L. plantarum *after infection by confocal laser scanning microscopy (CLSM), immunohistochemistry and Western blotting, the cytoskeleton protein F-actin were observed with FITC-phalloidin.

**Results:**

This study demonstrated that the transepithelial electrical resistance (TER) step down and dextran integrated intensity (DII) step up with time after infected with EIEC, but after treating with *L. plantarum*, the changes of TER and DII were improved as compared with EIEC group. *L. plantarum *prevented the damage of expression and rearrangement of Claudin-1, Occludin, JAM-1 and ZO-1 proteins induced by EIEC, and could ameliorate the injury of cytoskeleton protein F-actin infected with EIEC.

**Conclusion:**

*L. plantarum *exerted a protective effect against the damage to integrity of Caco-2 monolayer cells and the structure and distribution of TJ proteins by EIEC infection.

## Background

The intestinal epithelium forms a relatively impermeable barrier between the lumen and the submucosa. This barrier function is maintained by a complex of proteins composing the tight junction (TJ) that is located at the subapical aspect of the lateral membranes. The tight junctional complex comprises a large number of membrane-associated and membrane proteins, the latter including occludin, junction adhesion molecule (JAM), and claudins [1-4], which are responsible for forming the physical connections between cells that confer the basic barrier properties. These proteins are considered to be involved in the regulation of paracellular permeability. The TJ effect can be documented by reduction in transepithelial electrical resistance (TER). Some bacterial pathogens manipulate the apical-junctional complex from the apical surface. The cellular cascade induced in Enteropathogenic *Escherichia coli (*EPEC) infection, which leads to decrease in TER, is not well understood. One such strategy is to target the regulatory elements of the actin cytoskeleton. EPEC infects the apical surface of intestinal epithelial cells and modifies the actin cytoskeleton by forming actin-rich pedestals beneath the attached bacteria, firmly anchoring the bacterium to the host cell [5]. Changes in the host cell actin cytoskeleton could lead to a loss of absorptive surfaces in intestinal epithelial cells and account for the persistent diarrhea often associated with EPEC infection. Control of perijunctional actin may be also the final effector mechanism in modulating paracellular permeability [6].

It is increasingly recognized that *Lactobacillus plantarum (L. plantarum) *has the ability to protect against EPEC-induced damage of the epithelial monolayer barrier function by preventing changes in host cell morphology, attaching/effacing (A/E) lesion formation, monolayer resistance, and macromolecular permeability [7-10]. In recent years, Moorthy G et al [11] evaluated the effect of L. rhamnosus and L. acidophilus on the maintenance of intestinal membrane integrity during S. dysenteriae 1-induced diarrhea in rats. They found that induced rats showed a significant reduction in the membrane-bound ATPases and reduced expression of TJ proteins in the membrane, coupled with their increased expression in the cytosol, indicating membrane damage. Transmission electron microscopic studies correlated with biochemical parameters. Pretreatment with combination of L. rhamnosus and L. acidophilus significantly prevented these changes. However, the cellular mechanism involved in this protective effect still remained to be clarified.

The aim of this study was to investigate the molecular mechanisms underlying the beneficial effects of the *L. plantarum*. Moreover, as infections with Enteroinvasive *Escherichia coli *(EIEC) were accompanied by the disruption of epithelial integrity was also asked whether the presence of *L. plantarum *would influence the otherwise deleterious barrier disruption of caco-2 cells caused by EIEC bacteria. The permeability, the distribution and expression of tight junction proteins (such as Claudin-1, Occludin, JAM-1 and ZO-1) and the cytoskeleton were examined when infected with EIEC or adhesived of *L. plantarum *after infection.

## Results

### *L. plantarum *attenuates EIEC-induced decrease in TER of Caco-2 cells

**Figure 1:**
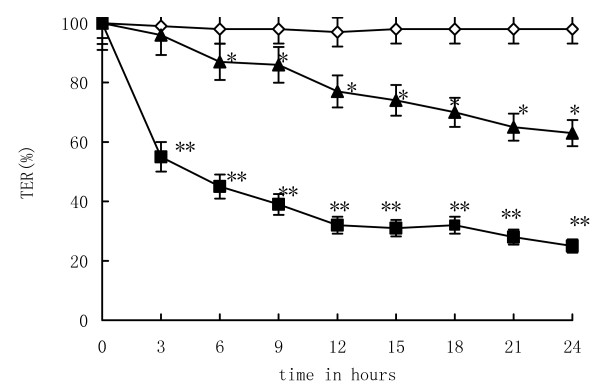
***L. plantarum *attenuates EIEC-induced decrease in TER of Caco-2 cells**. (◇) represented control group, (■) EIEC group, (▲) *L. plantarum *group. TER after enteroinvasive *E. coli *(EIEC) infection was significantly lower than the control after cultured 6 hours during 24 hrs. Each point represented the mean value obtained from 10 to 12 individual Caco-2 monolayers. Error bars showed the standard error. One-way ANOVA was performed with Tukey Kramer post-hoc comparison. * vs control group at different time, P < 0.05; ** vs *L. plantarum *group at different time, P < 0.05.

One complementary polarized epithelial cell lines (Caco-2) was used to assess barrier function in response to EIEC infection in the absence or presence of *L. plantarum*. TER of caco-2 monolayers were maintained 480 Ω·cm^2 ^after being cultured for 7 days. This was in contrast to caco-2 cells infected with EIEC which resulted in an approximately 46.67% decrease of TER from 480 Ω·cm^2 ^to 256 Ω·cm^2^. However, when Caco-2 cells were co-incubated simultaneously with EIEC and *L. plantarum*, the reduction of TER was 39.58% from 480 Ω·cm^2 ^to 290 Ω·cm^2^. The Caco-2 cells infected with EIEC induced to a substantial decrease of TER to 62.6% of the control values within 24 h (Fig. 1.).

### *L. plantarum *inhibits increases in macromolecular permeability of Caco-2 cells in response to EIEC infection

**Figure 2:**
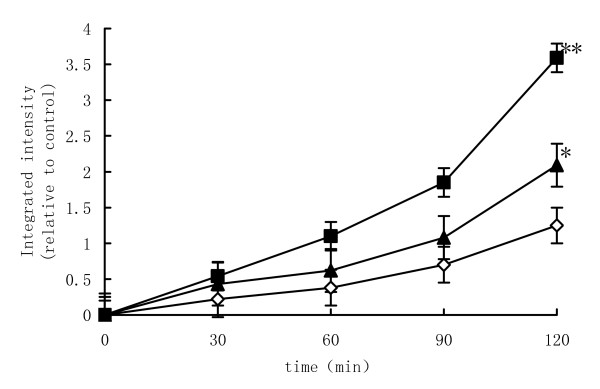
***L. plantarum *inhibits increases in macromolecular permeability of Caco-2 cells in response to EIEC infection**. Macromolecular permeability assays with Caco-2 cell monolayers using an infrared sensitive dextran (10-kDa) probe. (◇)represented control group, (■) EIEC group, (▲) *L. plantarum *group. Dextran integrated intensity after EIEC infected was significantly increased than the control group after cultured 60 min during 120 min. One-way ANOVA was performed with Tukey Kramer post-hoc comparison. * vs control group, P < 0.05; ** vs *L. plantarum *group, P < 0.05.

Macromolecular permeability assays with Caco-2 cell monolayers using an infraredsensitive dextran (10-kDa) probe (as measured by the signal intensity for basal medium samples) from apical to basolateral Transwell compartments (relative integrated intensity [RI] compared to control group, 1.25 ± 0.44, n = 4) demonstrated that EIEC-infected monolayers exhibited a marked increase in the permeability to the dextran probe (RI = 3.59 ± 0.51; n = 4) as compared with control group and *L. plantarum *group (RI = 2.09 ± 0.45; n = 4), P < 0.01 and P < 0.05, respectively. EIEC-induced increases in the dextran permeability of Caco-2 cell monolayers were reduced when epithelial cells were treated with *L. plantarum*, P < 0.05 (Fig. 2.).

### *L. plantarum *prevents EIEC-induced redistribution of Claudin-1, Occludin, JAM-1 and ZO-1 proteins

**Figure 3:**
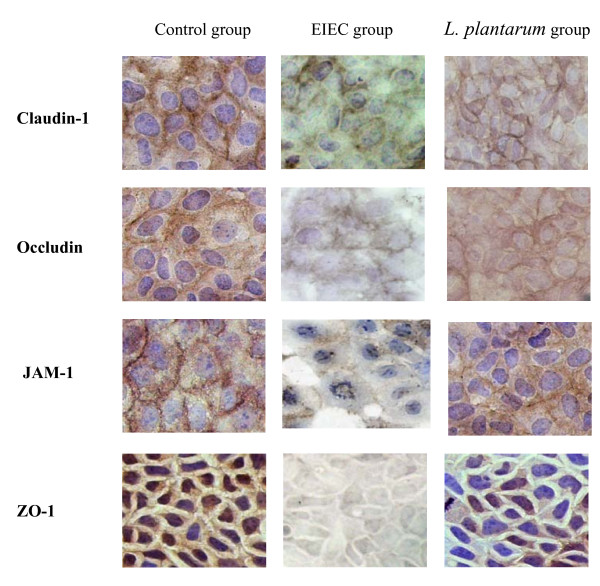
***L. plantarum *prevents EIEC-induced redistribution of Claudin-1, Occludin, JAM-1 and ZO-1 proteins**. Expression of TJ proteins (Claudin-1, Occludin, JAM-1, ZO-1) by immunohistrochemistry. Images shown were representative of at least 5 regions observed on the same slide, and 2 different sections were analyzed for each condition. Results were based on a double-blinded experiment.

TJ barrier function can also be affected by changes in the distribution of specific tight junctional proteins or their levels of expression. TJ were located between the adjacent Caco-2 cells, TJs associated proteins were continuously distributed with bright brown spots along membrane of the cells. The Claudin-1, Occludin, JAM-1 were located the outer of the membrane, ZO-1 protein was distributed in the cytoplasmic, their borders were very clear in the control group. In the caco-2 infected with EIEC, the expression of TJs associated-protein were decreased and the degradation developed in the EIEC group. In the co-incubation with *L. plantarum*, the brown spots distribution were decreased compared with control group, however, its expression were better than in EIEC group (Fig. 3.).

### *L. plantarum *prevents EIEC-induced expression of Claudin-1, Occludin, JAM-1 and ZO-1 proteins

**Figure 4:**
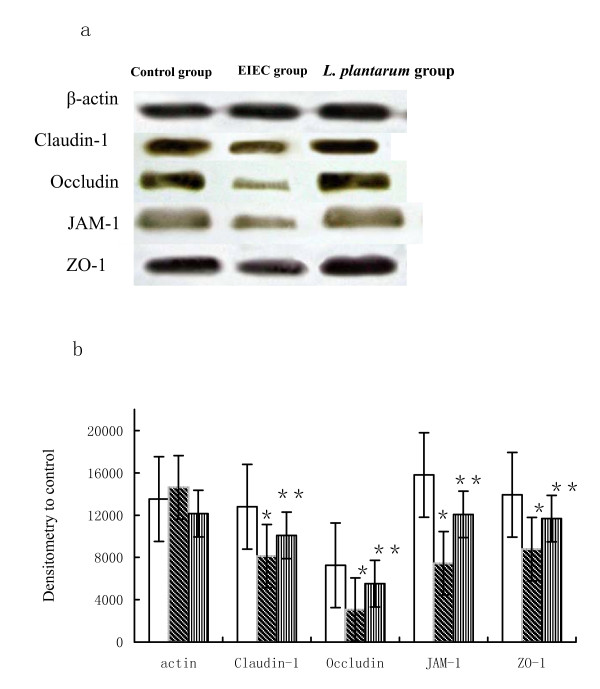
***L. plantarum *prevents EIEC-induced expression of Claudin-1, Occludin, JAM-1 and ZO-1 proteins**. (a) Western blotting analysis of Claudin, Occludin, JAM-1 and ZO-1 proteins. EIEC infection triggered a marked dissociation of the interactions between TJ proteins. Expression was analysed in membrane fractions by immunoblotting and subsequent densitometry. (b) The statistical evaluation of densitometric data represented protein expression of the three separate experiments (in percentage of all controls on the same blot). (□) control group, (▧) EIEC group, (▥) *L. plantarum *group. * vs control group, P < 0.05. ** vs EIEC group, P < 0.05. One-way ANOVA was performed with Tukey Kramer post-hoc comparison. Values were calculated by Student's *t*-test. All data are given as means (SE).

Western blot analyses were performed to determine the relative protein expression of Ocludin, Claudin, JAM-1 and ZO-1 in Caco-2 cells after treatment with EIEC and with *L. plantarum*. The intensity measurements for whole-cell proteins were determined from the ratio of the integrated intensity of the Ocludin, Claudin, JAM-1 and ZO-1 band to the integrated intensity of the β-actin band in the same sample. Western blotting of epithelial whole-cell protein extracts showed that TJ proteins expression were reduced in EIEC-infected cells compared to control group, P < 0.05. There were increased of the TJ proteins expression density in *L. plantarum *group as compared with EIEC group, P < 0.05 (Fig. 4A. and Fig. 4B.).

### *L. plantarum *prevents EIEC-induced rearrangements of Claudin-1, Occludin , JAM-1 and ZO-1 proteins

**Figure 5:**
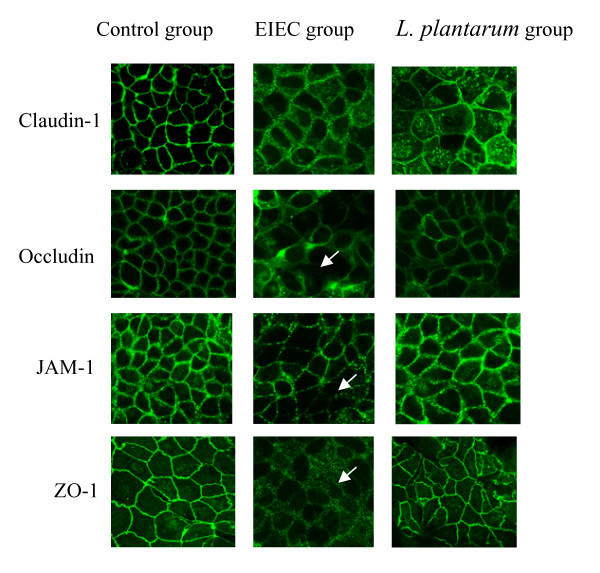
***L. plantarum *prevents EIEC-induced rearrangements of Claudin-1, Occludin, JAM-1 and ZO-1 proteins**. The intensity of the stain of the infected cells was decreased compared to that observed for control cells. In addition, areas where the TJ proteins belts were disrupted were present (arrows). Images were collected in 1-μm increments beginning at the apical aspect of the monolayers and optically sectioning to the basolateral membrane. Original magnification ×2400.

Confocal imaging was also performed to assess distribution of the TJs after exposure to EIEC. TJ associated proteins were continuously distributed with bright green spots along membrane of the cells. The Claudin-1, Occludin, JAM-1 were located the outer of the membrane, ZO-1 protein was distributed in the cytoplasmic, their borders were very clear in the control group. In the control Caco-2 intestinal monolayers, both ZO-1 and occludin were present at the apical intercellular borders in a belt-like manner, encircling the cells and delineating the cellular borders. In the infected caco-2, the green fluorescence were dispersedly distributed, and occludin staining became punctate with some loss from the membrane as opposed to the uniform membrane staining in controls. In the co-incubation with *L. plantarum*, the green spots distribution were decreased compared with control group, however its expression were better than in EIEC group (Fig. 5.).

### *L. plantarum *prevents EIEC-induced rearrangements of the epithelial cell cytoskeleton elements F-actin

**Figure 6:**
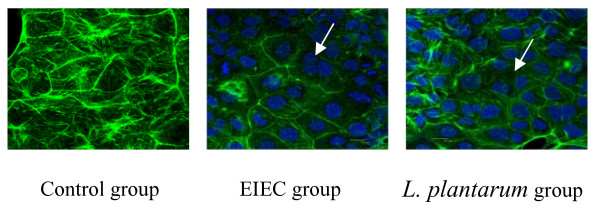
***L. plantarum *prevents EIEC-induced rearrangements of the epithelial cell cytoskeleton elements F-actin**. The intensity of the stain of the infected cells was decreased compared to that observed for control cells. In addition, the belts were disrupted were present (arrows). Original magnification ×2400.

To examine whether the barrier disruption is associated with redistribution of actin, F-actin staining with FITC-labelled phalloidin was carried out in the study. In the following studies, the possible involvement of cytoskeletal elements actin and the effect of *L. plantarum *on actin were visualized by fluorescent labeling of these structures. The staining pattern of control Caco-2 cells showed a continuous lined distributing at the cell borders and cytoskeletal. A high density of actin filaments was present at the apical peri-junctional regions and encircled the cells in a belt-like manner. In contrast, the type of the actin architecture in EIEC group showed disorganized and disrupted. The incubation of Caco-2 monolayers infected with EIEC resulted in a centripetal retraction of the peri-junctional actin filaments with separation of actins from the apical cellular borders. The EIEC-induced alteration of peri-junctional actin filaments was reversed by the re-introduction of *L. plantarum *(Fig. 6.).

## Discussion

Although many clinical studies have reported that probiotics, such as *L. plantarum*, have beneficial health effects [12-15], it is still difficult to ascertain their direct mechanism(s) of action. Therefore, the current trend in research in this field is to determine the mechanisms by probiotic are efficacious in treating specific gut abnormalities or protect against defined microbial infections [16].

Probiotics are reported to exert their beneficial effects by producing bacteriostatic or bactericidal agents [17,18], competitively excluding pathogenic bacteria [9], or regulating immunomodulatory effects [19,20]. Johnson-Henry KC et al [10] reported that with probiotic pretreatment there was corresponding attenuation of the Enterohemorrhagic Escherichia coli (EHEC) O157:H7-induced drop in electrical resistance and the increase in barrier permeability assays. L. rhamnosus GG protected epithelial monolayers against EHEC-induced redistribution of the claudin-1 and ZO-1 TJ proteins. Resta-Lenert S et al [20] hypothesized that probiotics and/or commensals could also reverse epithelial damage produced by cytokines. They found that deleterious effects of TNF-α and IFN-γ on epithelial function were prevented by probiotic, and to a lesser extent, commensal pretreatment. A Janus kinase (JAK) inhibitor synergistically potentiated effects of Streptococcus thermophilus (ST)/Lactobacillus acidophilus (LA) or Bacteroides thetaiotaomicron (BT) on TER and permeability, but p38, ERK1, 2, or PI3K inhibition did not. Finally, only probiotic-treated epithelial cells exposed to cytokines showed reduced activation of SOCS3 and STAT1,3. These data extended the spectrum of effects of such bacteria on intestinal epithelial function and may justify their use in inflammatory disorders. In addition, Seth A et al [21] evaluated the effect of Lactobacillus rhamnosus GG-produced soluble proteins (p40 and p75) on the hydrogen peroxide-induced disruption of TJ and barrier function in Caco-2 cell monolayers. Pretreatment of cell monolayers with p40 or p75 attenuated the hydrogen peroxide-induced decrease in TER and increased in inulin permeability in a time- and dose-dependent manner. p40 and p75 also prevented hydrogen peroxide-induced redistribution of occludin, ZO-1, E-cadherin, and beta-catenin from the intercellular junctions and their dissociation from the detergent-insoluble fractions. Both p40 and p75 induced a rapid increased in the membrane translocation of PKCbetaI and PKCepsilon. The attenuation of hydrogen peroxide-induced inulin permeability and redistribution of TJ proteins by p40 and p75 was abrogated by Ro-32-0432, a PKC inhibitor. p40 and p75 also rapidly increased the levels of phospho-ERK1/2 in the detergent-insoluble fractions. U0126 (a MAP kinase inhibitor) attenuated the p40- and p75-mediated reduction of hydrogen peroxide-induced TJ disruption and inulin permeability. These studies demonstrated that probiotic-secretory proteins protected the intestinal epithelial TJs and the barrier function from hydrogen peroxide-induced insult by a PKC- and MAP kinase-dependent mechanism.

This study broadens our current understanding of how probiotics exert their beneficial effects and emphasizes the ability of *L. plantarum *(CGMCC 1258) to protect polarized epithelial cells against the effects of *E. coli*-induced changes in barrier function. This study demonstrated that EIEC (O124:NM, ATCC 43893) disrupted epithelial TJ structure, including claudin-1, occludin, JAM-1, and ZO-1 distribution in Caco-2 culture cells, resulted in decreased TER and increased permeability to macromolecules. Infection models used by other investigators demonstrated that both probiotic mixtures (such as VSL#3) and additional single strains (e.g., *E. coli *Nissle 1917 and *L. casei *DN-114 001) prevented ZO-1 redistribution in response to *Salmonella enterica *serovar Dublin and enteropathogenic *E. coli *infections in vitro [23,23]. In our study, *L. plantarum *ameliorated the pathogen-induced redistribution of claudin-1, occludin, JAM-1, and ZO-1. We also demonstrated, for the first time, using confocal laser scanning microscopy, that *L. plantarum *treatment stabilized cellular TJs, thereby prevented EIEC (O124:NM, ATCC 43893)-induced redistribution of the integral TJ proteins.

To support microscopy observations, we also employed Western blotting techniques to determine levels of claudin-1, Occludin, JAM-1, and ZO-1. In contrast to EIEC infections, co-incubation with *L. plantarum *resulted in a close association of the TJ proteins with the cytoskeleton and a concentration of these proteins at the cellular contact sites that is known to stabilize TJ structures and helps to maintain the cell morphology of caco-2. In addition, we found that *L. plantarum *leaded to an increase expression of these proteins as had been shown by immunofluorescence and Western blotting experiments. These results demonstrated that the amount and localization of these TJ proteins appeared to be crucial for the beneficial effects of *L. plantarum*. Interestingly, co-incubation experiments of Caco-2 cells with both *L. plantarum *and EIEC simultaneously demonstrated that *L. plantarum *abrogated the detrimental effects of EIEC. When compared with the probiotic effect of *Lactobacillus acidophilus *(strain ATCC4356) investigated in a previous study by Resta-Lenert and Barrett [24] that showed that only the pretreatment but not the simultaneous exposure of epithelial cells with *L. acidophilus *prevents the invasion of an enteroinvasive *E. coli *strain (EIEC O29:NM), this demonstrated an extended activity of the probiotic EcN. In addition, our study showed that *L. plantarum *maintained the structure and rearrangement of the actin cytoskeleton, reversed the EIEC which leaded the F-actin cytoskeleton injury. A significant improvement in permeability was accompanied by disruption of the perijunctional F-actin.

## Conclusion

Taken together, we expanded findings of previous investigators by demonstrating that *L. plantarum *treatment interrupted the infectious processes of EIEC. By demonstrating the mode of action of this probiotic strain in attenuating EIEC infection, we expanded our knowledge regarding the protective contributions of this probiotic bacterium when it is cultured with epithelial cells. Accordingly, it is important to better define how individual probiotics elicit their beneficial effects as biotherapeutic agents against pathogen-induced disorders of the gastrointestinal tract.

## Methods

All reagents were obtained from Sigma (St Louis, MO, U.S.A.) unless otherwise indicated.

### Preparation of bacteria

*L. plantarum *strain CGMCC No.1258, a gift from Dr. Hang Xiaomin (Institute of Science Life of Onlly, Shanghai Jiao Tong University, Shanghai, China), was maintained on MRS agar (Difco Laboratories, Detroit, MI, U.S.A.). The bacteria were then grown overnight at 37°C in static nonaerated Dulbecco's modified Eagle medium (DMEM; Life Technologies, Gaithesburg, MD, U.S.A.) and 5% MRS agar (Difco), centrifuged, washed, and resuspended in cold Dulbecco's phosphate buffered saline (Life Technologies) to obtain a final concentration of 1.0 × 10^10^/mL. Quantification of bacterial suspension was determined using a standard curve for visible absorbance (600 nm; Beckman DU-50 spectrophotometer) compared with LBP colony-forming units (data not shown).

### Enteroinvasive Escherichia coli

EIEC strain 0124:NM (ATCC 43893, serotype O124:NM,) was a gift from (Shanghai CDC, China). They were grown overnight in static nonaerated DMEM, centrifuged, washed, and resuspended at a final concentration of 1.0 × 10^9^/mL. Quantification of bacterial suspension was determined using a standard curve for visible absorbance (600 nm; Beckman DU-50 spectrophotometer) compared with EPEC colony-forming units (data not shown).

### Preparation of monolayer

Caco-2 cells (human colonic epithelial-like cancer cell line obtained from the Cell Institute Affiliated China Science Research Institute, Shanghai, China) were grown in DMEM, containing 1% nonessential amino acids, 10% fetal bovine serum, 100 U/mL penicillin, 100 μg/mL streptomycin, and 0.25 μg/mL amphotericin B at 37°C in a humidified atmosphere with 5% CO_2_. The cells were plated at a density of 2 × 10^5 ^on a 0.4-μm pore cell culture insert with a diameter of one square centimeter (Costar/Corning, Corning, NY, U.S.A.) and allowed to reach confluency.

### Infection of intestinal epithelial monolayer

Caco-2 cells were washed three times in Hank's balanced salt solution (Life Technologies) to remove the antibiotic media. For rapid infection of the monolayer, 100 μL EIEC at 1.0 × 10^9^/mL was added to the apical side of the cell culture insert, and the insert was placed in a 50-mL tube and centrifuged at 200 *g *for 4 minutes. *L. plantarum *(100 μL of 1.0 × 10^10^/mL) was added to the monolayers in different groups for 24 hours. Caco-2 cells monolayers were cultured and served as the control group, Caco-2 cells were infected EIEC as the EIEC group, Caco-2 cells infected EIEC were co-incultured with *L. plantarum *as the *L. plantarum *group. The average number of Caco-2 cells in each monolayer was approximately 1 × 10^6^. The inoculation ratio of EIEC to Caco-2 cells was 100:1. The ratio of lactobacillus to EIEC was 10:1.

### Transepithelial electrical resistance (TER) and dextran permeability

Monolayers of Caco-2 cells were grown in filters (Millicell culture plate inserts; 0.4 μm pore size; 0.6 cm^2^). At full confluence (15–18 days), monolayers achieve a TER of >450 Ωcm^2 ^and was measured using a voltmeter (Millicell-ERS; Millipore, U.S.A.). The integrity of the confluent polarized monolayers was checked by measuring TER at different time intervals after treating with outer membrane proteins. TER (Ωcm^2^) = (Total resistance – Blank resistance) (Ω) × Area (cm^2^). Because TER values often vary among individual Caco-2 cultures, the electrical resistance value was recorded for each membrane before and after experimental treatment, and the percentage decrease from baseline (%TER) was calculated for each membrane.

Monolayers was assayed using a macromolecular conjugate probe, Alexa Fluor 647 dextran (10 kDa; Molecular Probes, Eugene, OR) [25]. Briefly, 0.2 ml of conjugated dextran suspended in DMEM (Invitrogen) was added to the apical compartment of Transwells, and 0.4 ml of DMEM alone added to the basolateral compartment. After incubation for 5 h at 37°C, samples (0.5 ml) from the basolateral compartment were placed into a 96-well plate (Corning) and analyzed to determine their fluorescent intensity using the Odyssey infrared imaging system (LI-COR Biosciences, Lincoln, NE) at a wavelength of 700 nm. Integrated intensities were expressed relative to the integrated intensity of medium samples from untreated controls.

### Expression of Claudin-1, Occludin, JAM-1 and ZO-1 by immunohistochemistry (IHC)

Monolayers of cells were prepared on glass coverslips, which were placed in six-well tissue culture plates (Corning Glass Works, Corning, N.Y.). After washing in PBS, permeabilization with 0.5% NP-40, and blocking of nonspecific binding sites with 5% normal goat serum (NGS). Preparations were fixed for 10 min at room temperature in 3.5% paraformaldehyde in PBS. Cell monolayers were incubated with a specific primary antibody for 30 min at room temperature, washed, and then incubated with the respective secondary antibody. Primary antibodies were diluted 1:20 to 1:100 (rabbit monoclonal anti-human Claudin-1, Occludin, JAM-1, ZO-1, Zymed, USA) in 2% bovine serum albumin-PBS. Secondary antibodies were goat anti-mouse immuno-globulin G (IgG) from Immunotech (Luminy, France) and were diluted 1:20 in 2% bovine serum albumin-PBS. Monolayers were then washed four times in saline and for 30 min and then color developed using diaminobenzidine solution. Monolayers were stained hematoxylin briefly after color development, and coverslips were mounted onto the slides using DPX medium (BDH Laboratories; Poole, UK).

### Fluorescence staining of Claudin-1, Occludin, JAM-1, ZO-1 and actin

Briefly, monolayers were fixed and permeabilized with methanol at -20°C and then incubated overnight at 4°C with primary antibodies against claudin-1, occludin (dilution 1:100, polyclonal rabbit anti-claudin-1 and anti-occludin antibody, Zymed, USA), JAM-1 and ZO-1 (dilution 1:50, polyclonal rabbit JAM-1 and anti-ZO-1 antibody, Zymed, USA), followed by a 2 h incubation with FITC-conjugated specific secondary antibody (Sigma) at room temperature (RT), in the dark. Subsequently, monolayers were washed several times with phosphate-buffered saline solution (10 mM PBS, pH 7.4, 136 mM NaCl, 2.6 mM KCl, 8.1 mM Na_2_HPO_4_, 1.4 mM KH_2_PO_4_), and then detached from the Anocell inserts and mounted with Vectashield (Vector Laboratories, Inc., Burlingame, CA). Cell staining was detected by confocal laser scanning microscopy (CLSM, Bio-Rad MRC 1024, Bio-Rad, Richmond, CA). To allow comparison between the treated and control groups, the microscopic examination of both groups was done in the same experimental session. Staining was absent from negative control inserts in which the primary antibodies were omitted. The degree of emitted fluorescence from the pancreas sections of the control and treated groups was measured using a software provided by the CLSM and expressed as arbitrary fluorescence units.

FITC-phalloidin staining was performed as previously described [26]. Caco-2 cells were treated with 60 μg of wild type EPEC OMP for 1 h. The treated monolayers were washed with PBS and fixed with 2% paraformaldehyde in PBS for 30 min. The fixed cells were then permeabilised with 0.1% Triton-X 100 in PBS for 5 min. The cells were washed thrice with PBS. They were then treated with 5 mg/ml of fluorescein isothiocyanate conjugated phalloidin in PBS for 30 min. After two washes in PBS to remove any trace of non-specific fluorescence, the cells were examined for cytoskeletal actin under a CLSM.

### Gel electrophoresis and western blotting

Monolayers of cells were collected immediately snap-frozen in liquid nitrogen. In preparation for SDS-PAGE, cells were thawed to 4°C. Cells were homogenized in chilled RIPA buffer (150 mM NaCl, 50 mM Tris-HCl, pH 7.4, 0.5% sodium deoxycholate, 1% Triton X-100, 1 mM EDTA), including protease and phosphotase inhibitors (1 mM PMSF, 1 mM Na_3_VO_4_, 1 mM NaF, and 5 g/ml of each of aprotinin, leupeptin, pepstatin). After centrifugation at 10 000 *g *for 10 min at 4°C, the supernatant was recovered and assayed for protein content (DC protein assay; Bio-Rad, Hercules, CA, USA). Equal amounts of total protein were separated on 10% SDS-polyacrylamide gels and then transferred to a nitrocellulose membrane. After blocking overnight in Tris-buffered saline (TBS) containing 0.05% Tween (TBS-T) and 5% dry powdered milk, membranes were washed three times for 5 min each with TBS-T and incubated for 2 h at room temperature in primary antibody (rabbit anti-Claudin-1, or rabbit antioccludin, or rabbit anti-JAM, or rabbit anti-ZO-1, both from Zymed Sigma). After three washes with TBS-T, the membranes were incubated for 1 h with horseradish peroxidase-conjugated secondary antibody. Following two washes with TBS-T and one wash with TBS, the membranes were developed for visualization of protein by the addition of enhanced chemiluminescence reagent (Amersham, Princeton, NJ, USA). Densitometric analysis was performed (Alpha Imager 1220 system) on three individual mice per treatment group.

### Statistical method

All experiments were done in triplicate and data represents mean and standard error. One-way ANOVA was performed on all experiments with Tukey Kramer post-hoc comparison. Significance was tested at P < 0.05. Densitometry was performed on immunoblots using a computer-assisted image analysis system (Quantity One, version 4.2.0; Bio-Rad, Hercules, CA, USA). Densitometry values are represented as the fold increase in densitometry compared to the values from uninfected control cells.

## Authors' contributions

ZWZ carried out the study, were responsible for data collection, sample analyses, and statistical analyses. XMH participated in the immunohistochmistry, fluorescence staining. YQJ participated in the gel electrophoresis and western blotting. All authors read and approved the final manuscript. HLQ conceived of the study, and participated in its design and coordination and helped to draft the manuscript, acquired the funding, wrote the original manuscript and edited all subsequent versions and final approved the manuscript.
